# Glucose-functionalized redox-responsive dihydroartemisinin prodrug nanosystem for targeted malaria therapy

**DOI:** 10.1016/j.ijpx.2025.100370

**Published:** 2025-07-31

**Authors:** Rongrong Wang, Jiaqi Yang, Jihong Qiang, Qingxia Li, Geng Wang, Canqi Ping, Kesheng Liu, Ruili Wang, Bin Zheng, Guolian Ren, Shuqiu Zhang

**Affiliations:** a School of Pharmacy, Shanxi Medical University, Taiyuan 030001, China; b Shanxi Provincial Key Laboratory of Drug Synthesis and Novel Pharmaceutical Preparation Technology, Shanxi Medical University, Taiyuan 030001, China; c Third Hospital of Shanxi Medical University, Taiyuan 030032, China; d The First Affiliated Hospital of Xi'an Jiaotong University, Xi'an 710061, China

**Keywords:** Dihydroartemisinin, Malaria, Redox-responsive prodrug nanoparticles, Glucose-targeted drug delivery

## Abstract

Although malaria has been effectively controlled, it still poses a threat to global health. Artemisinins are the first-line antimalarial drugs. However, their therapeutic efficacy is significantly limited by poor solubility and short biological half-life. To overcome these limitations and enhance drug accumulation in *Plasmodium*, we developed a glucose-functionalized redox-responsive dihydroartemisinin (DHA) prodrug nanosystem (D@GLU-PMs-SS). The nanosystem was prepared by using DHA-dithiodipropionic acid-octadecylamine prodrug and D-α-Tocopherol polyethylene glycol 1000 succinate-arbutin conjugate. The resultant D@GLU-PMs-SS exhibited excellent stability under conditions of storage and physiological environment. D@GLU-PMs-SS could be activated by glutathione (GSH), leading to the dissociation of nanoparticles and subsequent release of free DHA. *In vitro* experiments revealed that the host erythrocyte uptake of glucose-functionalized nanoparticles was significantly enhanced *via* GLUT-mediated transport. Cellular experiments illustrated that D@GLU-PMs-SS effectively reduced GSH concentrations in *Plasmodium*. Furthermore, D@GLU-PMs-SS displayed remarkable efficacy in inhibiting the growth of *Plasmodium* while maintaining biosafety. Overall, this study developed a strategy to enhance the targeting of nanoparticles to improve their therapeutic efficacy against malaria, warranting further investigation in clinical trials.

## Graphical abstract

D@GLU-PMs-SS was prepared through the nanoprecipitation method and subsequently administered intravenously to mice infected with *Plasmodium*. The nanoparticles were transported into the infective red blood cells and *Plasmodium* through glucose transporter (GLUT) and hexose transporter (HT). Following this, the prodrug nanoparticles degraded in the high glutathione (GSH) environment within *Plasmodium*, leading to the release of dihydroartemisinin (DHA). Ultimately, the intramolecular peroxide bridge of DHA generates reactive oxygen species (ROS) and depletes GSH levels, resulting in damage to the parasites.

**Figure:**
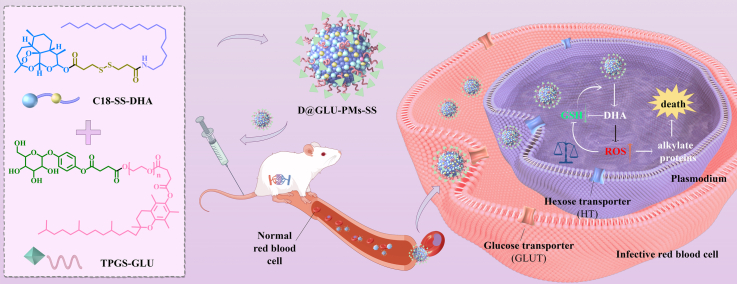

## 1 Introduction

Malaria continues to impose a substantial global health burden, particularly in tropical and subtropical regions (Poespoprodjo et al., 2023). According to the World Health Organization (WHO, 2024) statistics, malaria accounted for approximately 263 million cases and an estimated 597,000 fatalities in 2023. Artemisinin-based combination therapies (ACTs) remain the cornerstone of malaria treatment (Li et al., 2024a). Despite the concerning emergence of partial resistance to artemisinin in *Plasmodium falciparum* in certain regions, artemisinin derivatives continue to represent the most efficacious therapeutic options available, considering the limited alternative treatments (White and Chotivanich, 2024).

Artemisinin derivatives exert their antimalarial effects through the generation of free radicals *via* interaction with ferrous heme, leading to nonselective alkylation of *Plasmodium* proteins and subsequent cellular damage (Yang et al., 2020a). Additionally, these agents inhibit anaerobic glycolysis and perturb redox homeostasis, further exacerbating parasite damage (Gao et al., 2023a; Gao et al., 2023b; Gao et al., 2024). However, their efficacy is limited by poor solubility, a short half-life, and the emergence of resistance (Gérard Yaméogo et al., 2020; He et al., 2023; Zhu et al., 2022). Therefore, optimizing administration frequency and prolonging drug action during the trophozoite stage represent critical strategies for enhancing therapeutic outcomes.

Commercially available nanoformulations, such as liposomes, albumin-based nanoparticles, and polymer micelles, enhance drug uptake and prolong the duration of action (Barenholz, 2012; Hwang et al., 2020; Yardley, 2013). However, conventional nanocarrier systems face several challenges, including carrier toxicity, low drug loading, rapid release, and reduced efficacy, which necessitate the development of safer, high-capacity, and targeted platforms (Cai et al., 2015; Dai et al., 2011; Su et al., 2019). Prodrug nanomedicine, which integrates prodrug technology with nanodelivery systems, offers advantages such as simplified synthesis, defined structures, and high drug loading (Chien et al., 2023; Li et al., 2021). Notable examples include PTX-DHA, CP-4126, and CP-4055 (Bedikian et al., 2011; DiNardo CD et al., 2013; Stuurman et al., 2013). These formulations improve drug stability, bioavailability, and therapeutic efficacy by introducing long fatty chains. However, they often demonstrate limited therapeutic enhancement due to constrained drug release. To address this limitation, stimuli-responsive nanoformulations enable temporally controlled drug release at disease sites through enzymatic or microenvironmental triggers, thereby improving therapeutic efficacy (Kyu Shim et al., 2022).

The malaria parasite synthesizes reduced glutathione (GSH) and excretes oxidized glutathione (GSSG) into host erythrocytes, resulting in a higher intracellular GSH/GSSG ratio compared to host cells. This alteration facilitates the evasion of oxidative stress (Ginsburg et al., 2013; Mohring et al., 2017; Rocamora et al., 2018). The intracellular reducing microenvironment serves as a specific catalyst for drug release, enabling disulfide bonds (-SS-) to operate as responsive molecular switches. Concurrently, disulfide bonds can deplete GSH storage and induce synergistic oxidative damage, thereby increasing parasite vulnerability. Given these considerations, dihydroartemisinin (DHA) prodrug nanoparticles incorporating disulfide bonds as responsive module represent a promising therapeutic strategy for achieving temporally controlled drug release in *Plasmodium*.

To sustain rapid growth and reproduction, *Plasmodium* exhibits significant upregulation of the hexose transporter (HT) on its surface (Huang et al., 2021; Jiang et al., 2020). Additionally, the expression of glucose transporter (GLUT) is elevated on the membranes of host erythrocytes to accommodate the substantial glucose uptake required by the parasite (Heikham et al., 2015; Kirk, 2001; Mehta et al., 2005). The glucose uptake pathways in antimalarial therapy have garnered significant research interest, particularly regarding targeted therapeutic delivery. In this context, one promising approach involves functionalizing nanomedicine with glucose or other saccharides, which may enhance the spatial selectivity of *Plasmodium* for therapeutic drugs (Shafi et al., 2017).

In this study, we designed and constructed a nanoformulation of DHA to enhance its active targeting and therapeutic efficacy. DHA was combined with redox-responsive disulfide compounds and hydrophobic octadecylamine to create prodrug self-assembled nanoparticles. Subsequently, the surface of nanoparticles was functionalized with arbutin, a glucose structural analogue, to target the *Plasmodium* microenvironment. Upon entering the parasite and encountering high GSH concentrations, the disulfide bond undergoes reduction to sulfhydryl group. This hydrophilic sulfhydryl group facilitates hydrolysis of adjacent ester bond, thereby triggering the drug. The developed nanosystem demonstrated both effective targeting capabilities and favorable biosafety profiles. It effectively reduced GSH levels in malaria parasites, amplified the peroxidative damage effects of DHA, facilitated parasite clearance in *Plasmodium*-infected mice, and provided valuable insights for clinical applications.

## 2 Materials and methods

### 2.1 Materials

DHA and artesunate were procured from Chongqing Wuling Mountain Pharmaceutical, Kunming Pharmaceutical Group (Chongqing, China). The DHA prodrug (C18-SS-DHA) was synthesized following our group's established protocols. D-α-Tocopherol polyethylene glycol 1000 succinate (TPGS) was purchased from McLean Biochemical Technology Co., Ltd. (Shanghai, China). Succinic anhydride, octadecylamine, arbutin, N, N -dicyclohexylcarbodiimide (DCC), N -hydroxysuccinimide (NHS) and 4-dimethylaminopyridine (DMAP) were procured from Aladdin Biotechnology Co., Ltd. (Shanghai, China). Wright-Giemsa Stain Solution was obtained from Beijing Solarbio Science & Technology Co., Ltd. (Beijing, China). Coumarin-6 (C6) was acquired from Beijing Bailingwei Technology Institute (Wuhan, China). Hoechst 33342 was purchased from Thermo Fisher Scientific Institute (Shanghai, China).

### 2.2 Animals

A total of 164 male Institute of Cancer Research (ICR) mice, weighing between 18 and 22 g, were obtained from the Laboratory Animal Center at Shanxi Medical University for use in this study (SCXK(JIN)2019–004). All animal experiments adhered to the guidelines established by the Guide for the Care and Use of Laboratory Animals of Shanxi Medical University (Ethical approval number: 2019LL237). The mice were housed in cages at 25 °C ± 2 °C in a natural light–dark cycle, with free access to standard pelleted feed and drinking water throughout the experimental period. Mice used for all experiments were naive. No drug tests were done.

### 2.3 Malaria parasite

*Plasmodium yoelii* BY265 was generously provided by Professor Manyuan Wang (Capital Medical University, China).

### 2.4 Synthesis of dihydroartemisinin prodrug

The synthesis method of C18-SS-DHA have been published in our previous work (Fig. S1–5) (Wang et al., 2019). C18-CC-DHA was synthesized by forming an amide bond. Briefly, artesunate (0.42 mmol) was dissolved in dichloromethane (DCM) with NHS (0.50 mmol) and DCC (0.50 mmol). This mixture was maintained at 0 °C for 4 h, followed by a continuous reaction at room temperature for an additional 20 h. The resultant solution was then added dropwise to a DCM solution that contained octadecylamine (0.50 mmol). After 48 h, the resulting reaction mixture was purified using thin-layer chromatography (TLC). The identity of C18-CC-DHA was verified through fourier transform infrared spectroscopy (FTIR), proton nuclear magnetic resonance (^1^H NMR), carbon nuclear magnetic resonance spectroscopy (^13^C NMR) and high-resolution mass spectrometry (HR-MS).

### 2.5 Synthesis of D-α-Tocopherol polyethylene glycol 1000 succinate -succinic anhydride-arbutin (TPGS-GLU)

TPGS-GLU was synthesized through two-step reaction process. In the first step, TPGS (1.00 mmol), succinic anhydride (3.00 mmol), and DMAP (2.00 mmol) were dissolved in 10 mL of DCM in a round-bottomed flask. Subsequently, triethylamine (264 μL) was added dropwise to the reaction mixture and further stirred at 35 °C for 24 h. The mixture was then extracted three times with 1 % hydrochloric acid solution, after which the organic layer was washed three times with distilled water and dried over Na_2_SO_4_. The combined extracts were filtered and evaporated under reduced pressure to yield TPGS-COOH.

In the second step, TPGS-COOH (0.20 mmol) was dissolved in 2 mL of *N*,*N*-dimethylformamide. While stirring, NHS (1.60 mmol) and DCC (0.40 mmol) were added in order to activate the free carboxylic group of TPGS-COOH for further coupling with arbutin. The mixture was maintained at 0 °C for 6 h, followed by an additional 18 h at room temperature. A solution of arbutin (0.40 mmol) in *N*,*N*-dimethylformamide was added dropwise, followed by the addition of DMAP (0.40 mmol). The reaction proceeded at room temperature for 48 h. The product was extracted using 1 % hydrochloric acid solution and distilled water, followed by purification through dialysis. The chemical structure of the synthesized compounds was confirmed by FTIR, ^1^H NMR and ^13^C NMR.

### 2.6 Preparation of dihydroartemisinin prodrug nanoparticles

The glucose-functionalized redox-responsive nanoparticles (D@GLU-PMs-SS), glucose-free non-redox-responsive nanoparticles (D-PMs-CC), and glucose-functionalized non-redox-responsive nanoparticles (D@GLU-PMs-CC) were prepared through the nanoprecipitation method (Li et al., 2023; Yang et al., 2020b). In brief, the DHA prodrug (C18-SS-DHA or C18-CC-DHA, 10 mg) and a stabilizer (TPGS or TPGS-GLU, 2 mg) were dissolved in 1 mL of absolute ethanol and gradually added to 5 mL of deionized water under constant stirring. The organic solvent was subsequently removed by rotary evaporation under reduced pressure, yielding a nanoparticle suspension.

### 2.7 Characterization of nanoparticles

The particle size, polydispersity index (PDI), and zeta potential of D-PMs-CC, D@GLU-PMs-CC, and D@GLU-PMs-SS were measured using a Malvern Zetasizer Nano ZS90 particle size analyzer after suitable dilution. For morphological analysis, the nanoparticles were negatively stained with 1 % sodium phosphotungstate before analysis by transmission electron microscopy (TEM) using JEOL JEM-1200EX.

### 2.8 Evaluation of encapsulation efficiency and drug-loading capacity

The low-speed centrifugation method was employed to determine the encapsulation efficiency (EE) and drug loading capacity (DL) of D-PMs-CC, D@GLU-PMs-CC, and D@GLU-PMs-SS (Pi et al., 2022). In brief, nanoparticle suspensions were centrifuged at 3500 r/min for 20 min to separate the unencapsulated drugs. The concentration of encapsulated DHA prodrug in the supernatant and the total drug concentration in the formulation were determined by High-performance liquid chromatography with a post-column derivatization system (Wang et al., 2021). The EE and DL were calculated using the following eqs.

$$\mathrm{EE}=\frac{\text{Mass of the encapsulated drug}}{\text{Total mass of drug}}\times 100\%$$

$$\mathrm{DL}=\frac{\text{Mass of the encapsulated drug}}{\text{Total mass of nanoparticles}}\times 100\%$$

### 2.9 Stability evaluation

The dilution stability of nanoparticles was assessed by monitoring changes in particle size and PDI following dilution with distilled water at ratios of 2, 4, 8, 16, and 32 times. The storage stability of nanoparticles was evaluated by examining variations in particle size and EE over a 28-day storage period at 4 °C. The serum stability of nanoparticles was assessed by monitoring turbidity changes during incubation with an equivalent volume of fetal bovine serum at 37 °C for 4 h (Zuo et al., 2022).

### 2.10 *In vitro* release assay

The dynamic dialysis method was utilized to investigate the *in vitro* release profile of DHA from D-PMs-CC, D@GLU-PMs-CC, and D@GLU-PMs-SS. Briefly, 2 mL of nanoparticle suspension was loaded into a dialysis bag and immersed in 8 mL of 30 % (*v/v*) ethanol aqueous solution containing 1.5, 3.0, 5.0 mM of GSH. The experiment was conducted at 37 °C with constant stirring at 100 r/min. At the predetermined time intervals (0, 12, 24, 48, and 72 h), 200 μL of release medium was withdrawn and replenished with an equal volume of fresh release medium. The DHA concentration was measured using a UV spectrophotometric method. In brief, the DHA samples were dissolved in an assay solution composed of 2 % KOH and ethanol (1:4, *v/v*), and incubated at 60 °C for 30 min. The absorbance was measured at 289 nm.

### 2.11 Hemolysis experiments

To evaluate the blood compatibility of nanoparticles *in vitro*, a hemolytic assay was used to evaluate D-PMs-CC, D@GLU-PMs-CC, and D@GLU-PMs-SS samples (Li et al., 2024b). Rabbit red blood cells (RBCs) were collected and washed with saline until the supernatant appeared clear. The RBCs were then diluted with normal saline to obtain a 2 % (*v/v*) RBC suspension. Equal volumes of water, saline, D-PMs-CC, D@GLU-PMs-CC, and D@GLU-PMs-SS were incubated with 2 % RBC suspension for 3 h at 37 °C. After incubation, the samples were centrifuged at 1500 r/min for 15 min, and the absorbance of the supernatant was measured at 540 nm to calculate the hemolytic rate (HR) using the following eq.

$$\mathrm{HR}=\frac{A_{\text{sample}}-A_{\text{negative control}}}{A_{\text{positive control}}-A_{\text{negative control}}}\times 100\%$$

### 2.12 *In vivo* blood circulation of nanoparticles

The blood circulation duration of nanoparticles was performed using quanlitative analysis, with C6 as the fluorescence probe. Initially, D-PMs-CC, D@GLU-PMs-CC, and D@GLU-PMs-SS were co-assembled with C6 to prepare C6-PMs-CC, C6@GLU-PMs-CC, and C6@GLU-PMs-SS, respectively. Healthy and Plasmodium-infected ICR mice (infection ratio of 20 %) were injected with equivalent concentration of C6-sol, C6-PMs-CC, C6@GLU-PMs-CC, or C6@GLU-PMs-SS (*n* = 3 per group). Blood samples were collected at fixed time points (0.5, 1, 4, 7, 10, and 24 h). The blood samples were lysed using deionized water and methanol, followed by centrifugation at 12,000 r/min for 20 min to pellet cellular debris. The fluorescence intensity of the C6-containing supernatant was measured using a multimode microplate reader, with excitation and emission wavelengths of 460 nm and 500 nm, respectively.

### 2.13 *In vitro* cellular uptake of nanoparticles

To demonstrate the targeting ability of nanoparticles *in vitro*, C6 was used to label the nanoparticles. C6-PMs-CC, C6@GLU-PMs-CC and C6@GLU-PMs-SS were incubated with *Plasmodium-*infected red blood cells (iRBCs, infection ratio ≥ 20 %) in RPMI 1640 medium. Following a 2.5-h incubation, Hoechst 33342 was added to stain the *Plasmodium* nuclei. The samples were subsequently washed with PBS and fluorescence was analyzed using an ImageXpress Pico automated cell imaging analysis system (Molecular Devices).

To verify the role of GLUT in nanoparticle uptake, a GLUT inhibition assay was performed. Glcose (5 %), the natural ligand for GLUT, was added to the culture media. Following a 2.5-h incubation period and subsequent staining of *Plasmodium* nuclei with Hoechst33342, the intracellular localization of C6@GLU-PMs-CC and C6@GLU-PMs-SS was examined using the ImageXpress Pico automated cell imaging analysis system.

### 2.14 *In vivo* targeting ability of nanoparticles

C6-sol, C6-PMs-CC, C6@GLU-PMs-CC, and C6@GLU-PMs-SS were injected into both healthy and *Plasmodium*-infected mice (infection ratio of 20 %) (*n* = 3 per group). After 0.5, 1, and 4 h, whole blood was collected and centrifuged at 5000 r/min to isolate RBCs. The RBCs were washed twice with cold PBS and lysed with an equal volume of deionized water. Methanol was added to precipitate protein, followed by centrifugation. The fluorescence intensity of the supernatant was quantified using a microplate fluorescence reader.

### 2.15 Detection of intracellular reactive oxygen species (ROS)

Intracellular ROS levels were measured using 2′,7′-dichlorodihydrofluorescein diacetate (DCFH-DA) staining. In brief, iRBCs (infection ratio of 4 %) were seeded in 12-well black plates and treated with DHA solution (DHA-sol), D-PMs-CC, D@GLU-PMs-CC and D@GLU-PMs-SS for 24 h. The cells were washed twice with PBS and incubated with DCFH-DA at 37 °C for 30 min. After incubation, the cells were washed again and resuspended in ice-cold PBS. The fluorescence intensity was measured at excitation/emission wavelengths of 485/520 nm using a Varioskan™ LUX Multi-function microplate reader.

### 2.16 Measurement of GSH/GSSG level in Plasmodium

The GSH/GSSG levels in *Plasmodium* were determined using the Total Glutathione (T-GSH)/GSSG Colorimetric Assay Kit (Elabscience, Wuhan, China). Briefly, iRBCs with an infection ratio of 4 % were co-cultured with DHA-Sol, D-PMs-CC, D@GLU-PMs-CC and D@GLU-PMs-SS for 24 h. The iRBCs were lysed using saponin to extract the parasites, and each sample was divided into two aliquots. In one aliquot, intracellular GSSG was catalyzed to form reduced GSH, with 5,5-dithiobis (2-nitrobenzoic acid) (DTNB) employed to detect T-GSH. In the other aliquot, intracellular GSH was pre-excluded before GSSG catalysis, allowing the detection of GSSG. The GSH concentration was calculated by subtracting the GSSG concentration from the T-GSH concentration.

### 2.17 *In Vivo* antimalarial effect of nanoparticles

The antimalarial efficacy of DHA-sol, D-PMs-CC, D@GLU-PMs-CC, and D@GLU-PMs-SS was evaluated using a modified four-day suppression test (Lee et al., 2018). ICR mice were randomly assigned to 22 experimental groups (*n* = 5 per group). The normal group received no treatment, while the other groups were infected with 1.0 × 10^7^ *Plasmodium*-infected red blood cells from a donor animal. Two hours post-infection, mice were treated with saline (Control group), DHA-sol, D-PMs-CC, D@GLU-PMs-CC, and D@GLU-PMs-SS at doses of 1.1, 2.2, 4.4, 8.8, and 17.6 μmol/kg *via* intravenous injection for four consecutive days. 24 h after the final treatment, microscopic examination of Giemsa-stained blood smears was conducted to determine the infection and inhibition ratios. Additionally, the survival of mice in each group was monitored for 21 days. A more than 25 % reduction in body weight compared to the initial (day 0) body weight was considered the humane endpoint.

### 2.18 Hematology parameters, liver function indexes, and histological analysis

To assess the *in vivo* safety profile of nanoparticles, ICR mice were randomly divided into six groups (*n* = 3 per group). The normal group consisted of non-infected mice treated with saline. The remaining five groups were infected with parasites and administered saline, DHA-sol, D-PMs-CC, D@GLU-PMs-CC, and D@GLU-PMs-SS, respectively. The mice were conducted at the same time each day for four consecutive days. Following euthanasia two days after the final treatment, blood, liver, and spleen samples were collected for analysis of hematological parameters (WBC: white blood cell count, lymph: lymphocyte count, RBC: red blood cell count, HGB: hemoglobin, HCT: hematocrit, RDW: RBC distribution width), serum levels of liver enzymes (ALT: alanine aminotransferase, AST: aspartate aminotransferase), organ coefficients, and histopathological features using hematoxylin and eosin (H&E) staining.

### 2.19 Statistical analysis

Choice of sample size was based on studies published previously using similar animal models and experimental paradigms. Animals were assigned to experimental groups using a completely randomized design. The assignment of animal treatment or disposal was conducted randomly. The researchers responsible for collecting the baseline and outcome data were blind to randomisation until collection of all data was complete. No samples or animals were excluded from analysis. Experimental data were expressed as mean ± standard deviation. Statistical analyses were performed using SPSS software (version 22.0, IBM Corp., Armonk, NY, USA). A *P* < 0.05 was considered statistically significant.

## 3 Results and discussion

### 3.1 Synthesis of C18-CC-DHA

The synthetic pathway of C18-CC-DHA was illustrated in Fig.S6. The yield of C18-CC-DHA was determined to be 78 %. The FTIR, ^1^H NMR, ^13^C NMR and HR-MS results confirmed that the C18-CC-DHA was successfully synthesized.

The FTIR spectra of C18-CC-DHA was shown in Fig. S7. The band at 3295 cm^−1^ was contributed by the stretching vibrations of N—H. The absorption at 2916 cm^−1^ and 2849 cm^−1^ were attributed to the asymmetric and symmetric stretching vibrations of C—H in the long alkyl chain, respectively. The peak at 1749 cm^−1^was associated with the C=O stretching vibration. The bands at 1642 cm^−1^ and 1547 cm^−1^ were assigned as the amide I and amide II bands, respectively. Additionally, the peak at 1071 cm^−1^ was due to the C—O stretching vibration in cyclic ether.

^1^H NMR of C18-CC-DHA was shown in Fig. S8. ^1^H NMR (400 MHz, DMSO-D6) *δ* 7.84 (t, *J* = 5.6 Hz, 1H), 5.67 (d, *J* = 9.9 Hz, 1H), 5.56 (s, 1H), 3.02 (q, *J* = 6.9 Hz, 2H), 2.60 (td, *J* = 6.9, 3.9 Hz, 2H), 2.38 (t, *J* = 7.1 Hz, 2H), 2.30 (ddd, *J* = 9.8, 7.1, 4.4 Hz, 1H), 2.26–2.14 (m, 1H), 2.06–1.97 (m, 1H), 1.82 (ddd, *J* = 13.7, 6.5, 3.5 Hz, 1H), 1.62 (dtd, *J* = 14.0, 10.2, 3.8 Hz, 2H), 1.56–1.43 (m, 2H), 1.43–1.35 (m, 3H), 1.30 (s, 3H), 1.25 (s, 30H), 1.24–1.14 (m, 3H), 0.90 (d, *J* = 6.3 Hz, 3H), 0.89–0.84 (m, 3H), 0.78 (d, *J* = 7.3 Hz, 3H).

^13^C NMR of C18-CC-DHA was shown in Fig. S9. ^13^C NMR (151 MHz, CDCl_3_) *δ* 171.95, 171.23, 104.58, 92.28, 91.62, 80.22, 51.68, 45.35, 39.82, 37.41, 36.34, 34.21, 32.04, 31.89, 31.09, 29.99, 29.82, 29.79, 29.78, 29.74, 29.69, 29.48, 29.44, 27.03, 26.06, 24.71, 22.81, 22.12, 20.33, 14.24, 12.18.

HR-MS result of C18-CC-DHA was presented in Fig. S10. The theoretical accurate molecular mass of [C18-CC-DHA + Na]^+^ was calculated as *m/z* 658.4659, while the experimentally measured molecular mass was *m/z* 658.4652, with a relative error within ±3 ppm.

### 3.2 Synthesis of TPGS-GLU

To investigate the role of GLUT in iRBCs, a GLUT-targeted TPGS adduct (TPGS-GLU) was synthesized by conjugating TPGS and arbutin using succinic anhydride as the bridged linker. The synthetic process of TPGS-GLU, as depicted in Fig. S11, involved two key steps. First, TPGS underwent a ring-opening reaction with succinic anhydride to obtain TPGS-COOH. Subsequently, the hydroxyl group of arbutin reacted with the carboxyl group of TPGS-COOH to obtain the target compound, glucose-functionalized TPGS. The chemical structure of TPGS-COOH was confirmed by ^1^H NMR analysis. The chemical structure of TPGS-GLU was confirmed by FTIR, ^1^H NMR, ^13^C NMR.

^1^H NMR of TPGS-COOH is shown in Fig. S12. ^1^H NMR (400 MHz, DMSO‑*d*_6_) *δ* 12.38 (s, 1H), *δ* 4.26–4.04 (m, 4H), 3.72–3.43 (m, 84H), 2.88 (dd, *J* = 7.2, 4.8 Hz, 2H), 2.74–2.65 (m, 2H), 2.61–2.53 (m, 2H), 2.48–2.32 (m, 3H), 2.00 (s, 3H), 1.91 (d, *J* = 8.6 Hz, 6H), 1.82–1.62 (m, 2H), 1.50 (dt, *J* = 13.1, 6.6 Hz, 3H), 1.37 (s, 4H), 1.30–0.89 (m, 18H), 0.83 (dd, *J* = 9.6, 6.6 Hz, 14H).

The FTIR spectra for TPGS-GLU was presented in Fig. S13. The peak at 3316 cm^−1^ corresponds to the characteristic absorption peak of the O—H stretching vibration of arbutin, while the peak at 3084 cm^−1^ was attributed to the C—H stretching vibration of the aromatic ring. The peak at 2884 cm^−1^ represents the stretching vibration of C—H in the short alkyl chain, and the peak at 1100 cm^−1^ was associated with the C-O-C symmetric stretching vibrations of PEG.

^1^H NMR of TPGS-GLU is shown in Fig. S14. ^1^H NMR (400 MHz, DMSO‑*d*_6_) *δ* 7.27–6.48 (m, 3H), 5.32 (dd, *J* = 8.9, 3.4 Hz, 1H), 4.20–4.08 (m, 4H), 4.05–3.43 (m, 89H), 2.88 (dd, *J* = 7.4, 5.2 Hz, 2H), 2.77–2.63 (m, 2H), 2.60–2.52 (m, 4H), 2.48–2.30 (m, 3H), 2.00 (s, 3H), 1.90 (d, *J* = 8.7 Hz, 6H), 1.82–1.61 (m, 2H), 1.50 (dt, *J* = 13.1, 6.6 Hz, 3H), 1.42–1.32 (m, 4H), 1.32–0.93 (m, 21H), 0.83 (dd, *J* = 10.2, 6.5 Hz, 15H).

^13^C NMR of TPGS-GLU is shown in Fig. S15. ^13^C NMR (151 MHz, CDCl_3_) *δ* 172.89, 172.24, 172.05, 170.99, 149.45, 140.48, 126.71, 124.97, 123.03, 122.42, 118.53, 117.40, 116.01, 75.08, 70.58, 69.09, 68.61, 63.96, 63.82, 39.40, 37.56, 37.47, 37.41, 37.31, 32.81, 32.79, 32.72, 29.12, 28.86, 28.01, 24.83, 24.47, 22.77, 22.68, 21.07, 20.63, 19.80, 19.74, 19.70, 19.68, 19.64, 12.98, 12.12, 11.85.

### 3.3 Design, preparation and characterization of glucose-functionalized redox-responsive DHA prodrug nanoparticles

To enhance the spatiotemporal delivery efficacy of nanoparticles, we designed nanoparticles functionalized with glucose groups on their surface, which target and efficiently kill parasites *in vivo*. We selected glucose derivatives (arbutin) as the targeting moiety for iRBCs and parasites in this study. We hypothesized that due to the low expression of GLUT on the surface of normal RBCs, the probability of interaction between glucose-functionalized nanoparticles and normal RBCs would be insufficient to achieve a targeted accumulation effect. In contrast, iRBCs exhibit increased expression of GLUT on their surface (Izumo et al., 1989; Wei et al., 2018). Consequently, glucose-functionalized nanoparticles can be spatially and selectively distributed into iRBCs *via* GLUT. Furthermore, to sustain rapid growth and reproduction, *Plasmodium* exhibits high expression of hexose transporters on its surface (Slavic et al., 2011). This transporter facilitates the enhanced delivery of nanoparticles into *Plasmodium*, thereby improving spatial delivery efficiency. Upon entering *Plasmodium*, the elevated GSH/GSSG ratio compared to normal cells triggers the cleavage of disulfide bonds within the prodrug structure, followed by the subsequent cleavage of adjacent ester bonds (Najer et al., 2016). This process results in the release of DHA, increasing drug accumulation within *Plasmodium* and improving the temporal delivery efficiency of the nanoparticles.

All nanoparticles were prepared using a one-step nanoprecipitation technique. The nanoparticles are structured as a prodrug combining DHA and octadecylamine. The incorporation of a disulfide bond as the linking arm between DHA and octadecylamine prevents the premature release of DHA into the bloodstream and minimizes nonspecific drug distribution. The release of DHA is preferentially triggered in the presence of *Plasmodium*, which exhibits a high GSH/GSSG ratio. Additionally, the hydrophobic prodrug undergoes nanoself-assembly through hydrophobic interactions to form nanoparticles. The TPGS structure, with its vitamin E succinate hydrophobic end integrated into the nanoparticle framework and its polyethylene glycol (PEG) hydrophilic end oriented on the nanoparticle surface, enhances stability in aqueous solutions. Following the conjugation of glucose derivatives to the PEG end of TPGS, the glucose analogs are exposed outside the PEG coating, facilitating their targeting to iRBCs and *Plasmodium*, which exhibit high levels of sugar transporters. Furthermore, we synthesized DHA prodrug-loaded nanoparticles lacking a disulfide linker arm (D@GLU-PMs-CC) as a control for assessing GSH responsiveness and *in vivo* effectiveness. Additionally, we developed nanoparticles without a targeting moiety (D-PMs-CC) to serve as controls for studying both *in vitro* and *in vivo* cellular uptake in iRBCs.

**Fig. 1:**
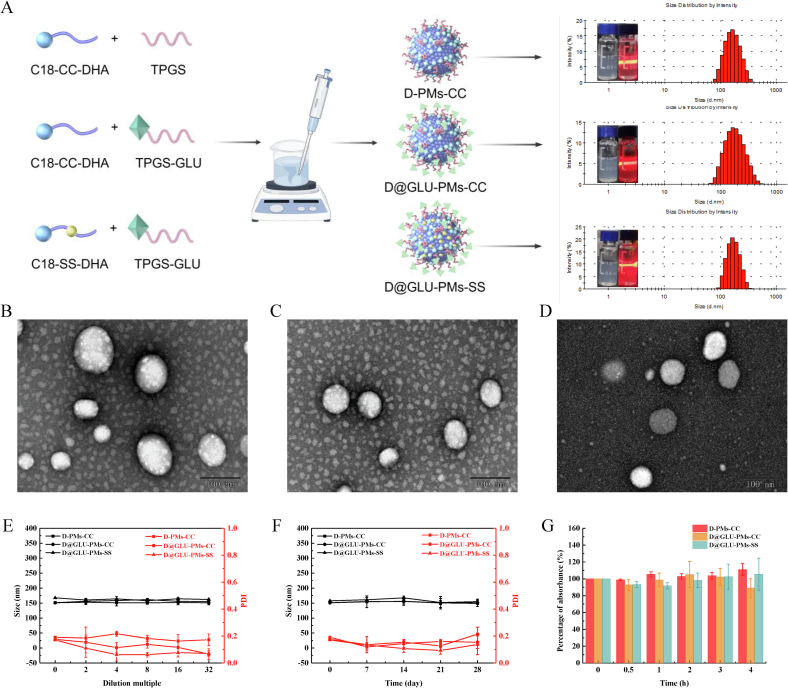
Preparation process and physicochemical characteristics of D-PMs-CC, D@GLU-PMs-CC and D@GLU-PMs-SS. Preparation process, visual appearance and particle size distribution of D-PMs-CC, D@GLU-PMs-CC and D@GLU-PMs-SS (A). TEM images of D-PMs-CC (B), D@GLU-PMs-CC (C) and D@GLU-PMs-SS (D) (scale bar: 100 nm). Changes in size and PDI of D-PMs-CC, D@GLU-PMs-CC and D@GLU-PMs-SS diluted with distilled water (E). Changes in size and PDI of D-PMs-CC, D@GLU-PMs-CC and D@GLU-PMs-SS in 28 days at 4 °C (F). Serum stability of D-PMs-CC, D@GLU-PMs-CC and D@GLU-PMs-SS(G). Data are presented as mean ± SD (*n* = 3).

DLS analysis showed that our formulation technique produced nanoparticles with an average diameter of approximately 160 nm and a narrow polydispersity index, as summarized in Fig. 1A and Table S1. TEM images displayed uniformly distributed spherical structures for D-PMs-CC, D@GLU-PMs-CC, and D@GLU-PMs-SS on the copper-coated carbon grid (Fig. 1B–D), highlighting the effectiveness of our method in generating consistent particle populations. Moreover, all three types of nanoparticles exhibited slightly negative surface charges (−18.93 ± 1.40 mV for D-PMs-CC, −20.97 ± 1.22 mV for D@GLU-PMs-CC, and − 10.09 ± 0.81 mV for D@GLU-PMs-SS), which prevent ion aggregation and enhance colloidal stability. Importantly, the prodrug-based nanoparticles functioned as carriers and active components, achieving remarkably high drug-loading efficiencies (88.96 % for D@GLU-PMs-SS, *w/w*, Table S2), which significantly exceed those of conventional nanoformulations (Bothiraja et al., 2009; Chen et al., 2018; Qiao et al., 2017; Xu et al., 2016). Additionally, the prepared nanoparticles contained only minimal TPGS without the inclusion of Tween 80 or ethanol, thereby minimizing the risk of adverse hypersensitivity reactions associated with conventional nanoformulations.

### 3.4 Stability of nanoparticles

The stability of the three prodrug nanoparticles under dilution was evaluated by monitoring changes in nanoparticle size and PDI under increasingly dilute conditions. The nanoparticles demonstrated remarkable dilution stability, showing minimal changes in diameter when diluted with distilled water at ratios of 2, 4, 8, 16, and 32 times (Fig. 1E). The storage stability of the nanoparticles at 4 °C was also studied. As depicted in Fig. 1F, there were no significant alterations in particle size or PDI for any of the three nanoparticle types over a 28-day period, indicating their excellent stability at low temperatures. Furthermore, the stability of the nanoparticles in serum was assessed through turbidity measurements. As illustrated in Fig. 1G, there was no significant increase in turbidity following the incubation of various nanoparticle suspensions with 50 % serum for 4 h. This result suggests that these nanoparticles do not promote aggregation caused by nonspecific interactions with serum proteins.

### 3.5 Reduction-triggered drug release

**Fig. 2:**
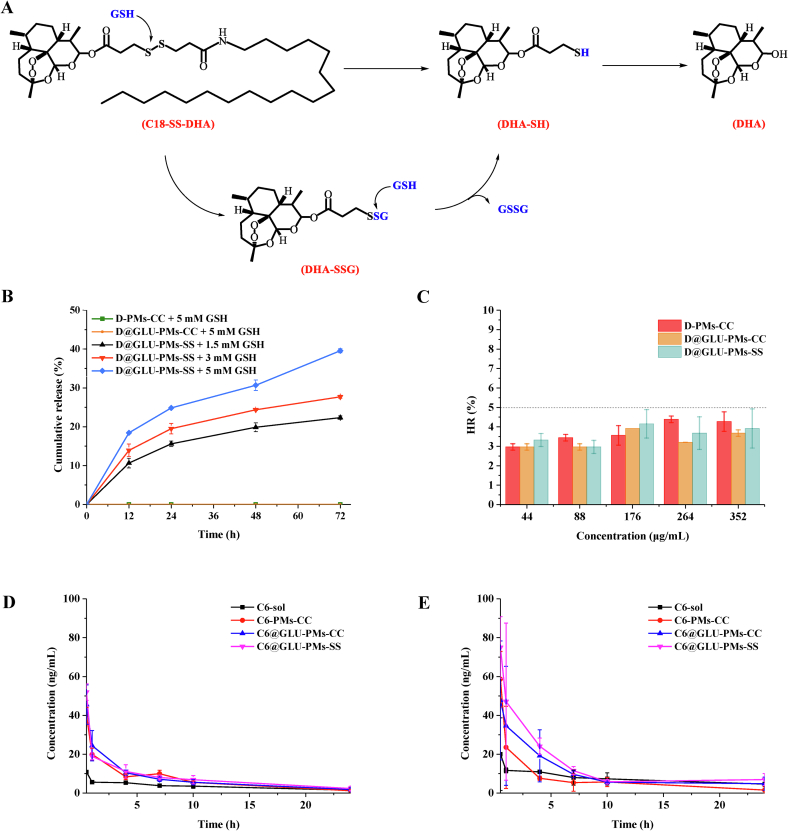
GSH-responsive mechanism of prodrug with disulfide bonds as linking arms (A). *In vitro* release profiles (B) and hemolytic rate (C) of D-PMs-CC, D@GLU-PMs-CC and D@GLU-PMs-SS. Blood circulation of C6-PMs-CC, C6@GLU-PMs-CC and C6@GLU-PMs-SS in healthy mice (D) and *Plasmodium*-infected mice (E). Data are presented as mean ± SD (*n* = 3).

The reduction-sensitive nature of disulfide bonds has been extensively documented in prior studies(Luo et al., 2016; Men et al., 2019; Mitra et al., 2012; Yang et al., 2021). As illustrated in Fig. 2A, we hypothesize that the presence of GSH facilitates the cleavage of disulfide bonds of D@GLU-PMs-SS, thereby generating thiol-containing intermediates (DHA-SH) and promoting the cleavage of adjacent ester bonds, which ultimately results in the release of DHA. Mass spectrometry confirmed the presence of the intermediates DHA-SH (Fig. S16). The *in vitro* drug release profiles of the nanoparticles were depicted in Fig. 2B. Both D@GLU-PMs-CC and D-PMs-CC demonstrated minimal hydrolysis when incubated in a release medium containing 5 mM GSH. In contrast, D@GLU-PMs-SS exhibited GSH-triggered drug release. Furthermore, as the concentration of GSH in the release medium increased, the rate of DHA release from D@GLU-PMs-SS progressively accelerated. These findings suggest that the disulfide bond in D@GLU-PMs-SS is redox-responsive, which may facilitate the release of DHA in the higher GSH concentration environment characteristic of *Plasmodium*.

### 3.6 Hemolysis experiments

Malaria is a systemic infectious disease caused by the *Plasmodium*. During the asexual infection phase, *Plasmodium* replicates within iRBCs. Intravenous administration of the drug allows it to enter the bloodstream directly, increasing the likelihood of direct interaction between *Plasmodium* and the medications. To assess the blood compatibility of D-PMs-CC, D@GLU-PMs-CC, and D@GLU-PMs-SS, the hemolysis of nanoparticles was further studied. As shown in Fig. 2C, the three types of nanoparticles did not induce significant hemolysis, with HR for D-PMs-CC, D@GLU-PMs-CC, and D@GLU-PMs-SS remaining below 5 %, meeting the safety requirements for intravenous injection.

### 3.7 *In vivo* blood circulation of nanoparticles

Hemolysis of red blood cells infected with *Plasmodium* represents the primary pathogenic mechanism observed during infection. This hemolytic reaction results in the release of heme from iRBCs into the systemic circulation, complicating the accurate quantification of artemisinin drugs levels in the bloodstream during malaria. In this context, C6-labeled nanoparticles were employed to indirectly assess the impact of nanonization on blood circulation(Ren et al., 2022; Zuo et al., 2022).

First, ICR mice were used as a model to investigate the *in vivo* blood circulation of C6-PMs-CC, C6@GLU-PMs-CC and C6@GLU-PMs-SS. As shown in Fig. 2D, C6 solution (C6-sol) showed rapid systemic clearance. In contrast, C6-labeled nanoparticles significantly prolonged the circulation time of C6. The area under the blood concentration curve (AUC_(0-t)_) for C6-PMs-CC (173.68 ± 13.38 h·μg·L^−1^), C6@GLU-PMs-CC (187.68 ± 22.53 h·μg·L^−1^) and C6@GLU-PMs-SS (201.90 ± 16.83 h·μg·L^−1^) was found to be greater than that of C6-sol (85.39 ± 6.43 h·μg·L^−1^).

Next, the blood circulation of the nanoparticles in *Plasmodium*-infected ICR mice was further evaluated (Fig. 2E). C6-sol was rapidly eliminated from the infected mice. In comparison, C6-PMs-CC exhibited a notable ability to reduce C6 clearance, which aligns with the findings observed in healthy ICR mice. Furthermore, the AUC_(0-t)_ for C6@GLU-PMs-CC (278.85 ± 96.80 h·μg·L^−1^) and C6@GLU-PMs-SS (343.03 ± 56.71 h·μg·L^−1^) was increased compared with that of C6-sol (166.27 ± 39.18 h·μg·L^−1^). Notably, both C6@GLU-PMs-CC and C6@GLU-PMs-SS demonstrated a marginally greater AUC compared to C6-PMs-CC (186.09 ± 29.38 h·μg·L^−1^). This enhancement is primarily attributed to the glucose groups modification on the nanoparticles surface, which increases their affinity for iRBCs, thereby elevating C6 levels throughout the blood circulation. Additionally, the amount of released C6 from C6@GLU-PMs-SS was greater than that from C6@GLU-PMs-CC. This finding is further supported by the presence of disulfide bonds in the prodrug structure, which enhances the colloidal stability of C6@GLU-PMs-SS (Graf et al., 2013; Sun et al., 2019; Wang et al., 2014), preserving the integrity of the nanostructure and reducing phagocytosis by the reticuloendothelial system.

### 3.8 Targeting ability of nanoparticles

**Fig. 3:**
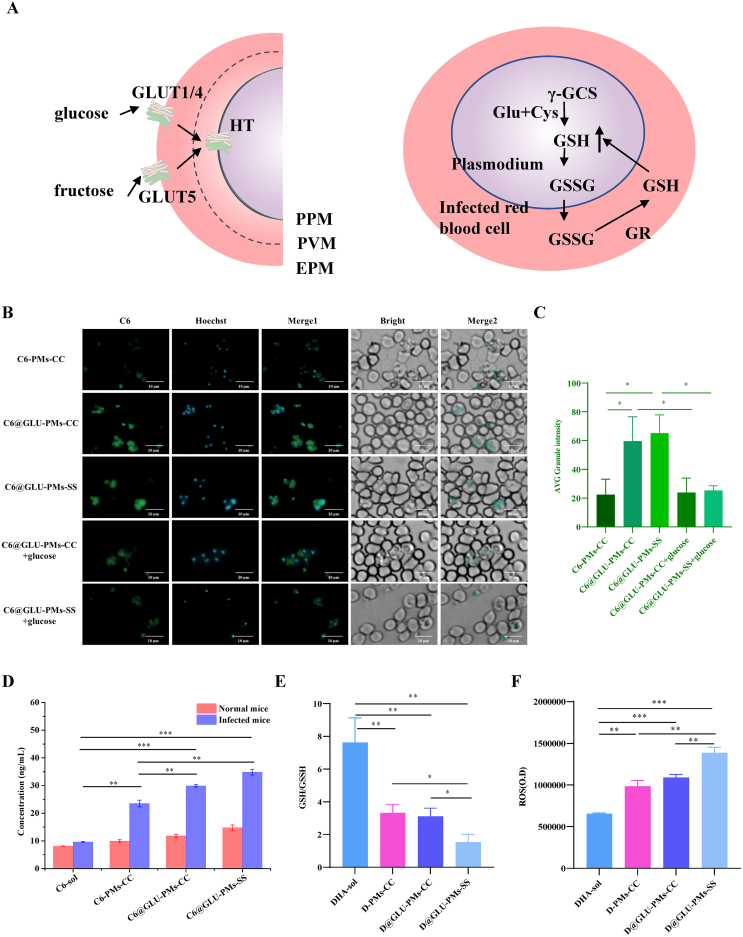
Schematics of sugar uptake (left) and biosynthesis and transport of GSH (right) in *Plasmodium* and its host cell (A). Fluorescence microscopy images of the *Plasmodium*-infected red blood cells co-incubated with C6-PMs-CC, C6@GLU-PMs-CC and C6@GLU-PMs-SS (scale bar: 10 μm) (B). The florescence intensity in (B) was further statistically quantified (C). The data are presented as means ± SD and were analyzed by one-way analysis of variance (ANOVA), followed by the *post hoc* LSD test (^⁎^*P* < 0.05, *n* = 3). The fluorescence intensity in red blood cells was examined at 0.5 h following the injection of C6-labeled nanoparticles into mice (D). The data are presented as means ± SD and were analyzed by independent samples *t* -test (^⁎^*P* < 0.05, ^⁎⁎^*P* < 0.01, ^⁎⁎⁎^*P* < 0.001, *n* = 3). The GSH/GSSG ratios (E) in parasites and the levels of ROS (F) in *Plasmodium*-infected red blood cells were analyzed following treatments with D-PMs-CC, D@GLU-PMs-CC, and D@GLU-PMs-SS. Data are presented as mean ± SD and were analyzed by independent samples t -test (^⁎^*P* < 0.05, ^⁎⁎^*P* < 0.01, ^⁎⁎⁎^*P* < 0.001, *n* = 3). PPM, parasite plasma membrane; PVM, parasitophorous vacuole membrane; EPM, erythrocyte plasma membrane. (For interpretation of the references to colour in this figure legend, the reader is referred to the web version of this article.)

We hypothesize that nanoparticles functionalized with glucose derivatives (C6@GLU-PMs-CC and C6@GLU-PMs-SS) can preferentially penetrate iRBCs mainly *via* specific GLUT transporters on the membranes of iRBCs (Fig. 3A left). To investigate this hypothesis, we utilized C6 as the delivery payload for the nanoparticles and demonstrated the targeted accumulation of glucose-functionalized nanoparticles using the ImageXpress Pico automated cell imaging analysis system. The data revealed that, compared to C6-PMs-CC, both C6@GLU-PMs-CC and C6@GLU-PMs-SS exhibited a significantly higher uptake into iRBCs (Fig. 3B). To validate the specificity of this interaction, we implemented competitive inhibition experiments by co-incubating the nanoparticles with glucose. Fluorescence imaging indicated a notable decrease in the green fluorescent dots within iRBCs following the introduction of glucose alongside C6@GLU-PMs-CC and C6@GLU-PMs-SS. Additionally, we quantified the intracellular localization level across different experimental groups using the ImageXpress Pico automated cell imaging analysis system, corroborating the trends observed in the microscopic fluorescence images (Fig. 3C). These results demonstrate that modifications with glucose derivatives significantly improve the targeting efficiency of nanoparticles toward iRBCs and facilitate the uptake of drug-laden nanoparticles.

*In vitro* experiments demonstrate that C6@GLU-PMs-CC and C6@GLU-PMs-SS effectively target iRBCs. Consequently, we examined the *in vivo* targeting capabilities of the prepared nanoparticles. As illustrated in Fig. 3D and Fig. S17, the uptake of nanoparticles in the RBCs of healthy ICR mice did not significantly increase, regardless of glucose derivative modifications, compared to free C6. In contrast, *Plasmodium* infection induces alterations in the morphology and function of the iRBC membrane, resulting in a marked increase in the uptake of all three types of nanoparticles in iRBCs compared to free C6. *Plasmodium* infection upregulates the expression of GLUT on the surface of host red blood cells. As a result, C6@GLU-PMs-CC and C6@GLU-PMs-SS exhibited enhanced accumulation in iRBCs compared to C6-PMs-CC and C6-sol (0.5 and 4.0 h after injection). These results presented a potent argument for modification of nanoparticles with glucose derivatives to enhance their antimalarial effects *in vivo*.

### 3.9 *In vitro* intracellular ROS and GSH-GSSG detection

To mitigate the toxic effects of ROS generated by immune responses, *Plasmodium* synthesizes GSH and effluxes GSSG into iRBCs to establish a GSH/GSSG redox system (Fig. 3A right) (Ginsburg et al., 2013; Zhao et al., 2024). Artemisinin drugs increase ROS levels in iRBCs by breaking the intramolecular endoperoxide bridges, resulting in protein damage and parasite death (Bridgford et al., 2018; Yang and Yang and Bell, 1994). Elevated levels of GSH have been reported to render parasites less sensitive to artemisinin drugs (J et al., 2015; Witkowski et al., 2017). Disulfide bonds are frequently incorporated into the design of GSH-sensitive prodrugs, which react with sulfhydryl groups in GSH to release active drugs while concurrently depleting GSH (Hadipour Moghaddam et al., 2018; Han et al., 2019; Iyer et al., 2020). To verify the effect of GSH-sensitive DHA prodrug nanoparticles on the redox microenvironment of *Plasmodium*, we measured the GSH/GSSG ratio in *Plasmodium* and ROS levels in iRBCs following treatment with various nanoparticles. As shown in Fig. 3E, the GSH/GSSG ratios in the D-PMs-CC and D@GLU-PMs-CC groups were significantly reduced by 56.53 % and 59.18 %, respectively, compared to that of DHA-sol. Excitingly, the introduction of disulfide bonds into the prodrug structure resulted in a 50.66 % reduction in the GSH/GSSG ratio of D@GLU-PMs-SS compared to D@GLU-PMs-CC. The ROS levels in iRBCs treated with D-PMs-CC, D@GLU-PMs-CC, and D@GLU-PMs-SS were significantly enhanced compared to DHA-sol (Fig. 3F). The ROS levels in the GSH-sensitive D@GLU-PMs-SS group were 2.11- and 1.27-fold higher than those in the DHA-sol and D@GLU-PMs-CC, respectively. In summary, the formulation of DHA into prodrug nanoparticles enhances the oxidative damage induced by DHA. The GSH-sensitive prodrug structure facilitates the consumption of GSH within *Plasmodium*, further compromising the parasite's oxidative defense mechanisms and intensifying the cell death induced by DHA.

### 3.10 *In vivo* antimalarial efficacy of nanoparticles

**Fig. 4:**
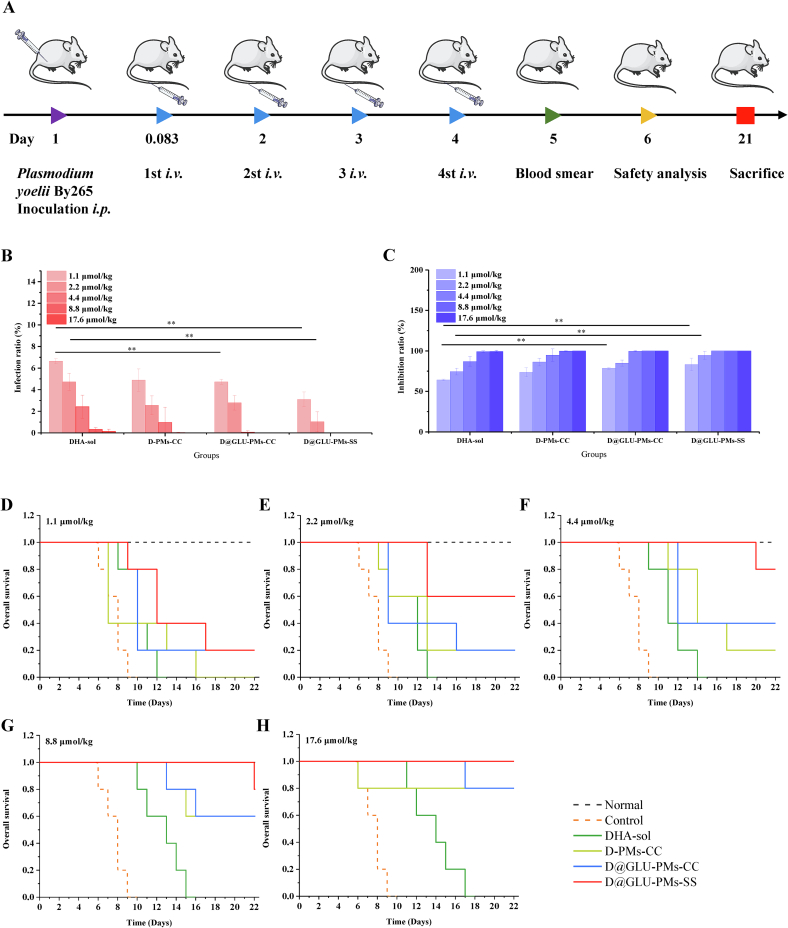
The schedule of establishment of *Plasmodium*-infected mice models and protocol of drug administration (A). Infection ratio (B) and inhibition ratio (C) of groups treated with various concentrations of DHA-sol, D-PMs-CC, D@GLU-PMs-CC, and D@GLU-PMs-SS on day 5. The data are presented as means ± SD and were analyzed by one-way analysis of variance (ANOVA), followed by the *post hoc* LSD test (homogeneity of variance) or Tamhane test (heterogeneity of variance) (^⁎⁎^*P* < 0.01, *n* = 5). Survival curves of mice treated with various formulations at doses of 1.1 μmol/kg (D), 2.2 μmol/kg (E), 4.4 μmol/kg (F), 8.8 μmol/kg (G), and 17.6 μmol/kg (H).

*In vivo* antimalarial efficacy of DHA-sol, D-PMs-CC, D@GLU-PMs-CC, and D@GLU-PMs-SS in *Plasmodium-*infected ICR mice was presented in Fig. 4. At the five concentrations tested, the infection ratios in the three nanoparticles groups and DHA-sol group were significantly lower than those in the control group (*P* ≤ 0.01, Fig. 4B). When administered at a dosage of 1.1 μmol/kg, the inhibition ratios observed in mice were 78.49 % ± 1.23 % for D@GLU-PMs-CC and 83.26 % ± 7.78 % for D@GLU-PMs-SS. In comparison, the inhibition ratio for DHA-sol at the same dosage was only 64.12 % ± 1.14 % (Fig. 4C). Moreover, no parasites were detected in the blood smears of mice from the D-PMs-CC (17.6 μmol/kg), D@GLU-PMs-CC (8.8 and 17.6 μmol/kg) and D@GLU-PMs-SS (4.4, 8.8 and 17.6 μmol/kg) groups after one day of drug withdrawal. These results indicate that DHA prodrug nanoparticles enhance the antimalarial efficacy of DHA.

Additionally, analysis of mice survival plots revealed differences between the control group and the drug treatment groups (Fig. 4D-H). In this experiment, all mice in the control group succumbed to severe parasitemia by day 9. In comparison to the control group, the five doses of the DHA-sol group demonstrated a more pronounced therapeutic effect, with all mice in this group succumbing by day 17. Compared to animals administered DHA-sol alone, those treated with D-PMs-CC, D@GLU-PMs-CC, and D@GLU-PMs-SS exhibited longer lifespans and higher survival rates. Notably, in the D@GLU-PMs-SS group, the survival rate exceeded 80 % at the three highest doses.

Based on the observed infection ratio, inhibition ratio, and survival rate, it can be inferred that D@GLU-PMs-SS enhances the antimalarial activity of DHA. Early research indicates that the therapeutic effects of DHA are limited by its short biological half-life. *In vitro* release studies indicated that the slow release of DHA from nanoparticles is expected to maintain effective DHA concentrations in parasites within high-glutathione environments. Cellular uptake experiments confirmed that the surface modification of nanoparticles with glucose derivatives enhances their targeting to iRBCs. In stark contrast to DHA-sol, these modifications confer significant advantages to D@GLU-PMs-SS, thereby enhancing its efficacy in eliminating parasites.

### 3.11 *In vivo* biological safety of nanoparticles

**Fig. 5:**
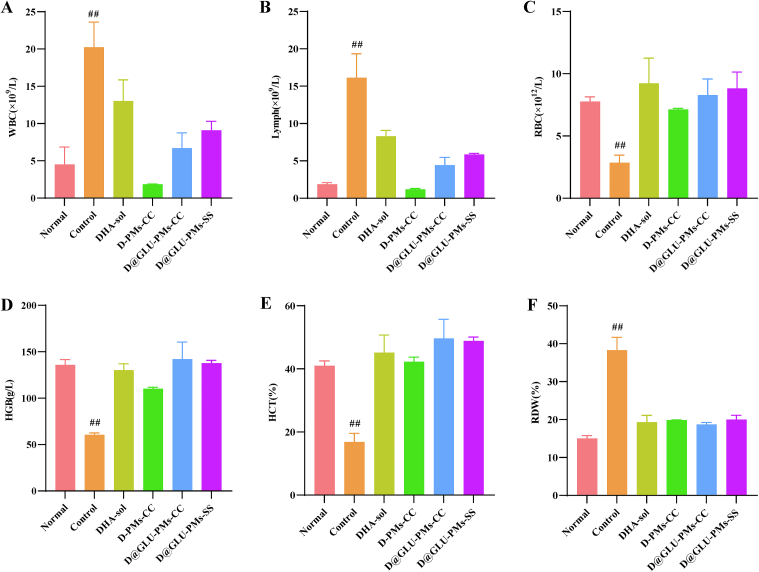
The blood routine parameters of WBC (A), Lymph (B), RBC (C), HGB (D), HCT (E), and RDW (F) of *Plasmodium*-infected mice treated with DHA-sol, D-PMs-CC, D@GLU-PMs-CC, and D@GLU-PMs-SS. The data are presented as means ± SD and were analyzed by independent samples t -test (^##^*P* < 0.01: Control *vs* all other groups, *n* = 3).

**Fig. 6:**
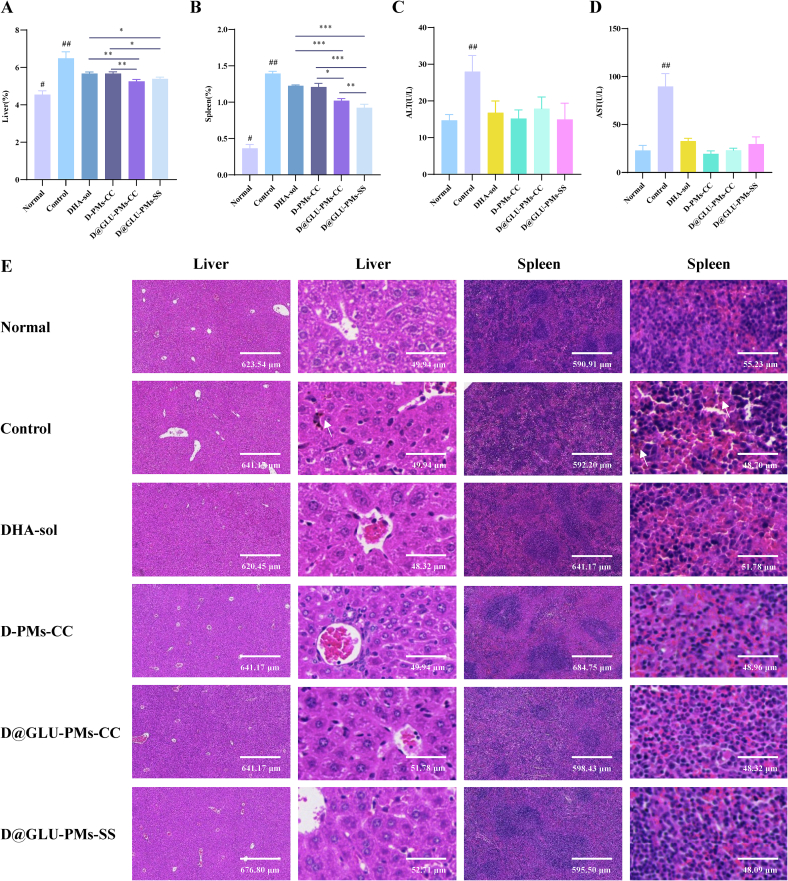
The liver coefficient (A), spleen coefficient (B), ALT (C), AST (D) of *Plasmodium*-infected mice treated with different preparations. The data are presented as means ± SD and were analyzed by independent samples t -test (^⁎^*P* < 0.05, ^⁎⁎^*P* < 0.01, ^⁎⁎⁎^*P* < 0.001, ^#^*P* < 0.05: Normal *vs* all other groups, ^##^*P* < 0.01: Control *vs* all other groups, *n* = 3). H&*E*-stained images of main organs from mice after following various treatments (E).

The *in vivo* safety of nanoparticles is an important criterion for clinical practice. To assess the safety of D-PMs-CC, D@GLU-PMs-CC, and D@GLU-PMs-SS *in vivo*, we measured hematological parameters, liver function indices, and histological analyses in all treatment groups. There was no statistical difference in hematological parameters among most groups, except for the control group (*Plasmodium*-infected mice treated with saline), which exhibited immune system dysregulation due to malaria infection (Fig. 5). The liver and spleen coefficients and blood biochemical indices were elevated in the control group, while treatment with DHA-sol reduced these indices (Fig. 6A-6D). Notably, the D@GLU-PMs-SS group exhibited the most significant decrease in liver and spleen coefficients. These results suggest that DHA prodrug nanoparticles do not induce systemic toxicity and may provide a protective effect on the liver and spleen.

In addition, we analyzed the histomorphological changes in the liver and spleen of *Plasmodium*-infected mice in various treatment groups. Only the liver sections from the control group showed inflammatory infiltration, extensive brown-yellow hemozoin deposition, and disordered arrangement of liver cells. The spleen sections from the control group showed a marked in white pulp with unclear structure and massive brown-yellow hemozoin deposition in the red pulp. In contrast, all other groups showed no signs of liver or spleen tissue injury (Fig. 6E). These results confirm that DHA treatment reduces liver and spleen damage caused by malaria infection. D-PMs-CC, D@GLU-PMs-CC and D@GLU-PMs-SS were found to be non-physiologically toxic and are therefore suitable as prodrug nanoparticles to enhance the therapeutic effects of DHA.

## 4 Conclusions

In this investigation, we successfully developed a GLUT-targeted, redox-responsive DHA prodrug delivery system (D@GLU-PMs-SS) for targeted malaria therapy using a prodrug strategy and nanoformulation. D@GLU-PMs-SS exhibited suitable particle sizes, enhanced stability, and GSH-responsive drug release profiles. Nanoparticles functionalized with the glucose derivative arbutin demonstrated specific accumulation in *Plasmodium*-infected red blood cells *via* GLUT-mediated transport, both *in vitro* and *in vivo*, demonstrating superior targeting efficiency compared to non-functionalized nanoparticles. GLUT inhibition experiments provided compelling evidence that D@GLU-PMs-SS enhanced intracellular accumulation through a GLUT-mediated mechanism. Moreover, by leveraging GSH-responsive drug release and active targeting enabled by glucose functionalization, D@GLU-PMs-SS achieved preferential accumulation within infected erythrocytes while significantly reducing off-target effects. In the *Plasmodium-*infected mouse model, D@GLU-PMs-SS demonstrated remarkable capabilities in inhibiting *Plasmodium* growth while ensuring biosafety. Given *Plasmodium*'s reliance on glucose for energy uptake, we developed a glucose-functionalized nanosystem. This innovative nanodelivery system is characterized by its simplicity, reproducibility, and scalability. Consequently, this approach could also be applied to the reformulation of other potential antimalarial agents that exhibit poor pharmacokinetics or bioavailability, thereby enhancing their targeting ability and therapeutic efficacy.

## CRediT authorship contribution statement

**Rongrong Wang:** Writing – review & editing, Writing – original draft, Project administration, Methodology, Investigation, Funding acquisition, Data curation. **Jiaqi Yang:** Writing – review & editing, Methodology, Investigation, Data curation. **Jihong Qiang:** Methodology, Investigation, Data curation. **Qingxia Li:** Validation, Methodology. **Geng Wang:** Validation, Methodology. **Canqi Ping:** Validation, Methodology. **Kesheng Liu:** Visualization, Methodology. **Ruili Wang:** Validation, Supervision, Investigation. **Bin Zheng:** Validation, Supervision, Investigation. **Guolian Ren:** Validation, Supervision. **Shuqiu Zhang:** Writing – review & editing, Writing – original draft, Visualization, Validation, Supervision, Funding acquisition, Data curation.

## Declaration of competing interest

The authors declare the following financial interests/personal relationships which may be considered as potential competing interests:(Graphical abstract and Fig. 1A left were created using Figdraw 2.0 (www.figdraw.com). Selected artwork (mice and syringe) shown in the Fig. 4A were adapted from images provided by Servier Medical Art (Servier; https://smart.servier.com/), licensed under a Creative Commons Attribution 4.0 Unported License. If there are other authors, they declare that they have no known competing financial interests or personal relationships that could have appeared to influence the work reported in this paper.)

## Data Availability

Data will be made available on request.

## Appendix A Supplementary data

Supplementary material Image 1
