# Supplementary material for: Glucose-functionalized redox-responsive dihydroartemisinin prodrug nanosystem for targeted malaria therapy
Source: Int J Pharm X. 2025 Jul 31;10:100370. doi: 10.1016/j.ijpx.2025.100370 (PMC12344195; doi:10.1016/j.ijpx.2025.100370)
Supplement: Supplementary file 1 — Supplementary material [file mmc1.docx]

Supporting Information

Glucose-functionalized redox-responsive dihydroartemisinin prodrug nanosystem for targeted malaria therapy

Rongrong Wang^#*, a, b, c^, Jiaqi Yang^#, a^, Jihong Qiang^a^, Qingxia Li^a^, Geng Wang^a,d^, Canqi Ping^a^, Kesheng Liu^a^, Ruili Wang^a, b^, Bin Zheng^a, b^, Guolian Ren^a, b^, Shuqiu Zhang^*, a, b^

^a^ School of Pharmacy, ^b^ Shanxi Provincial Key Laboratory of Drug Synthesis and Novel Pharmaceutical Preparation Technology, Shanxi Medical University, Taiyuan 030001, China

^c^ Third Hospital of Shanxi Medical University, Taiyuan, 030032, China

^d^ The First Affiliated Hospital of Xi'an Jiaotong University, Xi'an, 710061, China

^*^Corresponding author: Shuqiu Zhang; Rongrong Wang (School of Pharmacy, Shanxi Medical University, 56 Xinjian South Road, Taiyuan 030001, China)

E-mail addresses: shuqiu.zhang@126.com; shuqiu.zhang@sxmu.edu.cn; rongrongwang0213@126.com

^#^These authors contributed equally to this work.

Synthesis of C18-SS-DHA

The synthesis method of C18-SS-DHA has been published in our previous work (Wang et al., 2019). The synthetic pathway of C18-SS-DHA was illustrated in Fig. S1. In brief, a solution of 3,3-dithiobispropionic acid (0.95 mmol) in 3 mL of acetic anhydride was stirred at 30°C for 3.5 hours. Excess acetic anhydride was subsequently removed via rotary evaporation. Following this, a solution containing dihydroartemisinin (0.32 mmol) and 4-dimethylaminopyridine (0.03 mmol) in dichloromethane was added to the aforementioned product and allowed to react at 35°C for 48 hours. The resulting products were purified using thin-layer chromatography (TLC), yielding pure DHA–SS–COOH. Next, DHA–SS–COOH (2.10 mmol) was dissolved in 10 mL of dichloromethane and reacted with N -hydroxysuccinimide (2.50 mmol) and N, N -dicyclohexylcarbodiimide (2.50 mmol) at 0°C for 4 hours in a flask. The reaction was then allowed to proceed at room temperature for an additional 20 hours. In the final stages, octadecylamine (2.50 mmol) was added to the filtered solution, and the mixture was stirred at room temperature for 48 hours. The resulting solution was concentrated under reduced pressure and further purified using TLC.

The fourier transform infrared spectroscopy (FTIR), proton nuclear magnetic resonance spectroscopy (^1^H NMR), carbon nuclear magnetic resonance spectroscopy (^13^C NMR) and high-resolution mass spectrometry (HR-MS) results confirmed that the C18-SS-DHA was successfully synthesized.

FTIR of C18-SS-DHA was shown in Fig. S2. The peak observed at 3316 cm⁻¹ was associated with the stretching vibrations of the N–H bond. At 2916 cm⁻¹ and 2850 cm⁻¹, the absorptions correspond to the asymmetric and symmetric stretching vibrations of C–H within the long alkyl chain, respectively. The peak at 1748 cm⁻¹ was attributed to the stretching vibration of C=O. The bands observed at 1638 cm⁻¹ and 1533 cm⁻¹ were identified as amide I and amide II bands, respectively. Additionally, the peak noted at 1131 cm⁻¹ arises from the stretching vibration of C–O.

^1^H NMR of C18-SS-DHA was shown in Fig. S3. ^1^H NMR (400 MHz, DMSO-D6) δ 7.94 – 7.83 (m, 1H), 5.71 (d, J = 9.8 Hz, 1H), 5.59 (s, 1H), 3.07 – 3.00 (m, 2H), 2.98 – 2.93 (m, 2H), 2.91 (t, J = 7.1 Hz, 2H), 2.86 – 2.78 (m, 2H), 2.46 (t, J = 7.1 Hz, 2H), 2.38 – 2.26 (m, 1H), 2.26 – 2.15 (m, 1H), 2.05 – 1.97 (m, 1H), 1.83 (ddt, J = 13.5, 6.5, 3.5 Hz, 1H), 1.67 – 1.59 (m, 2H), 1.58 – 1.46 (m, 2H), 1.30 (s, 3H), 1.25 (s, 34H), 1.22 – 1.15 (m, 2H), 0.91 (d, J = 6.3 Hz, 3H), 0.89 – 0.85 (m, 3H), 0.80 (d, J = 7.1 Hz, 3H).

^13^C NMR of C18-SS-DHA was shown in Fig. S4. ^13^C NMR (151 MHz, CDCl_3_) δ 170.74, 170.71, 104.64, 92.40, 91.66, 80.23, 51.67, 45.35, 39.83, 37.42, 36.33, 35.97, 34.19, 34.17, 33.92, 32.86, 32.05, 31.89, 29.82, 29.78, 29.74, 29.72, 29.70, 29.48, 29.45, 27.07, 26.04, 24.71, 22.81, 22.13, 20.33, 14.24, 12.29.

HR-MS of C18-SS-DHA was shown in Fig. S5. The theoretical accurate molecular mass of [C18-SS-DHA + Na]^+^ was calculated as *m/z* 750.4413, while the experimentally measured molecular mass was *m/z* 750.4410, with a relative error within ± 3 ppm.


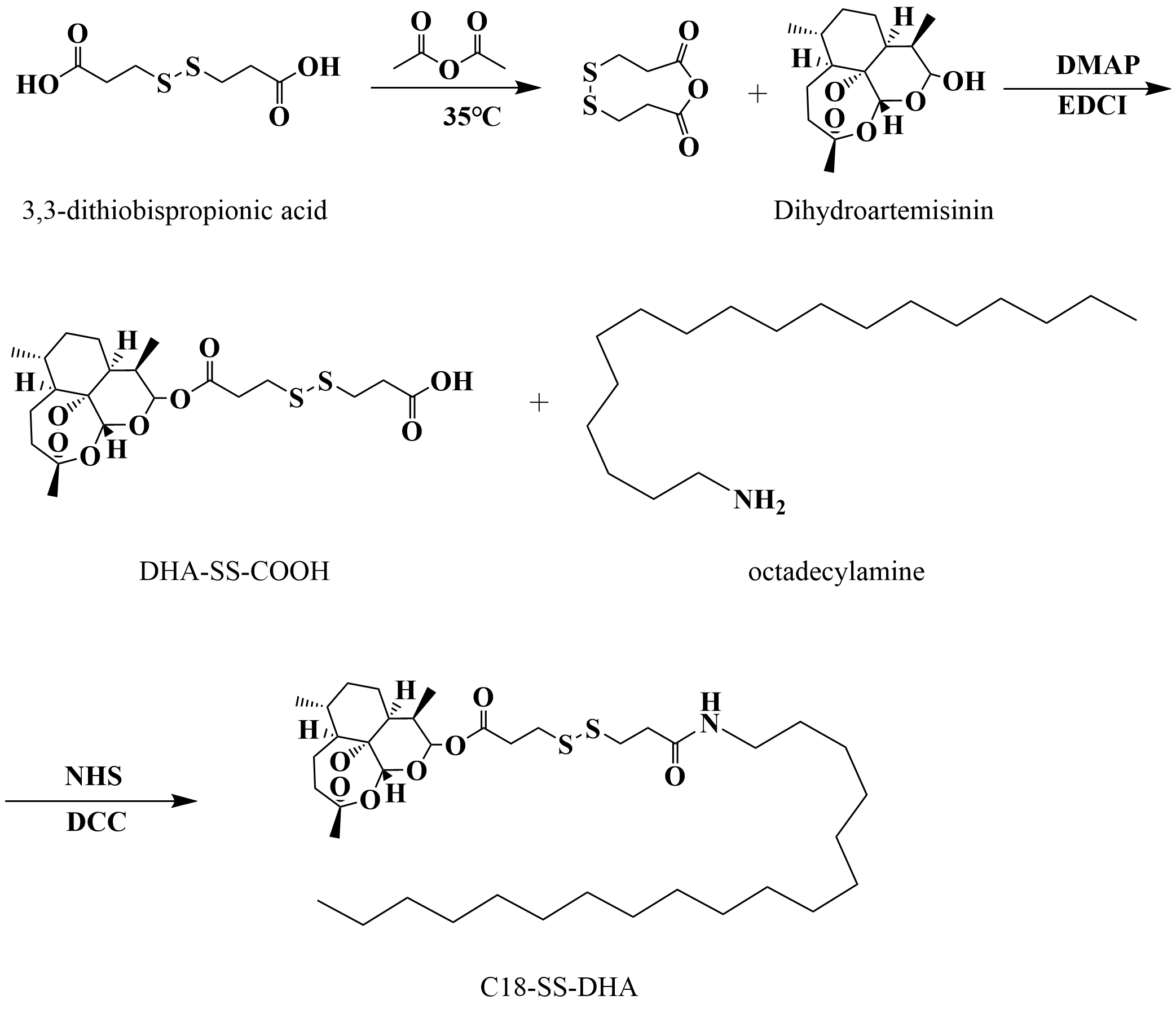


Fig. S1. Synthetic scheme of C18-SS-DHA.

Fig. S2. FTIR of C18-SS-DHA.

Fig. S3. ^1^H NMR of C18-SS-DHA.


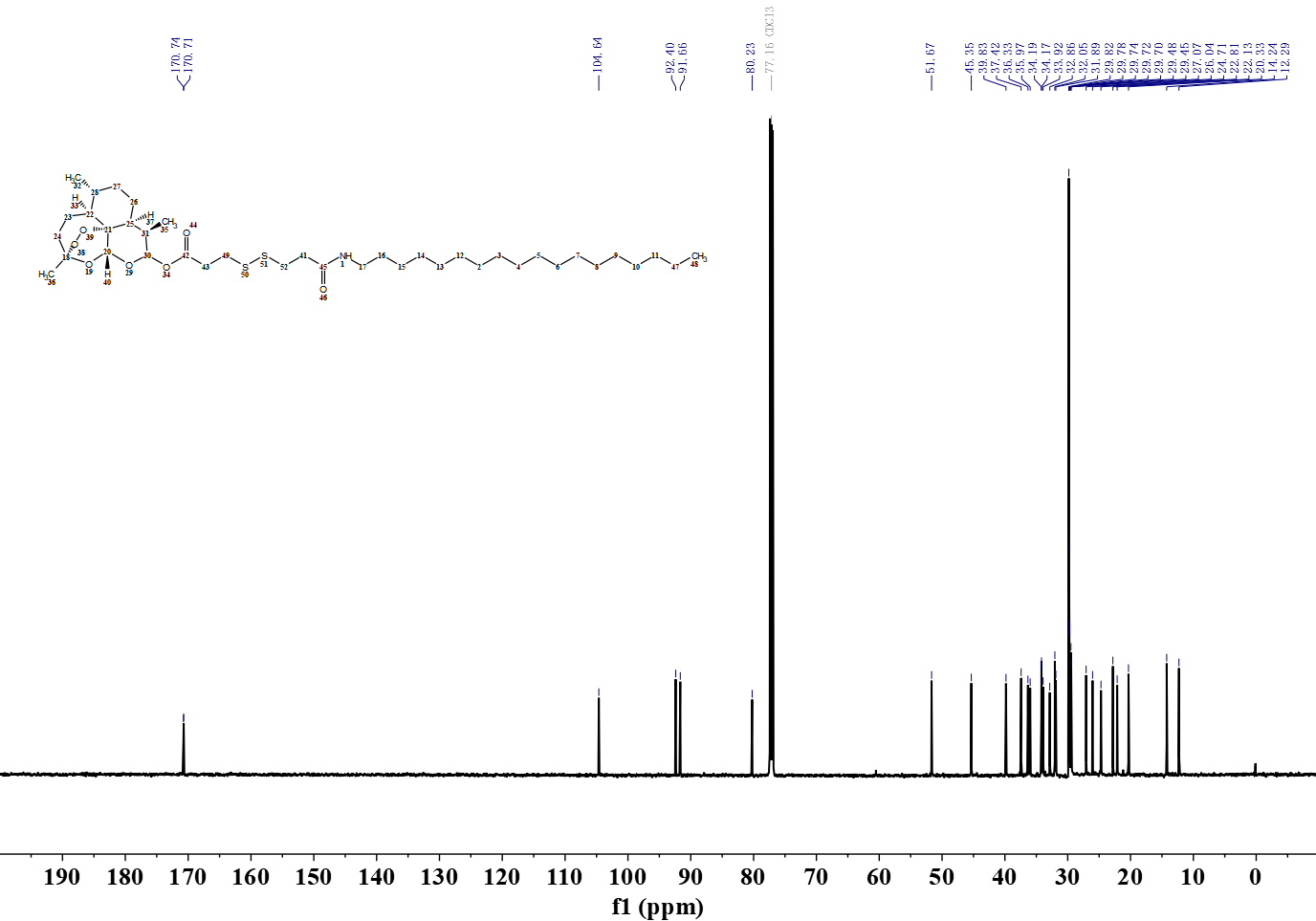


Fig. S4. ^13^C NMR of C18-SS-DHA.

Fig. S5. HR-MS of C18-SS-DHA.


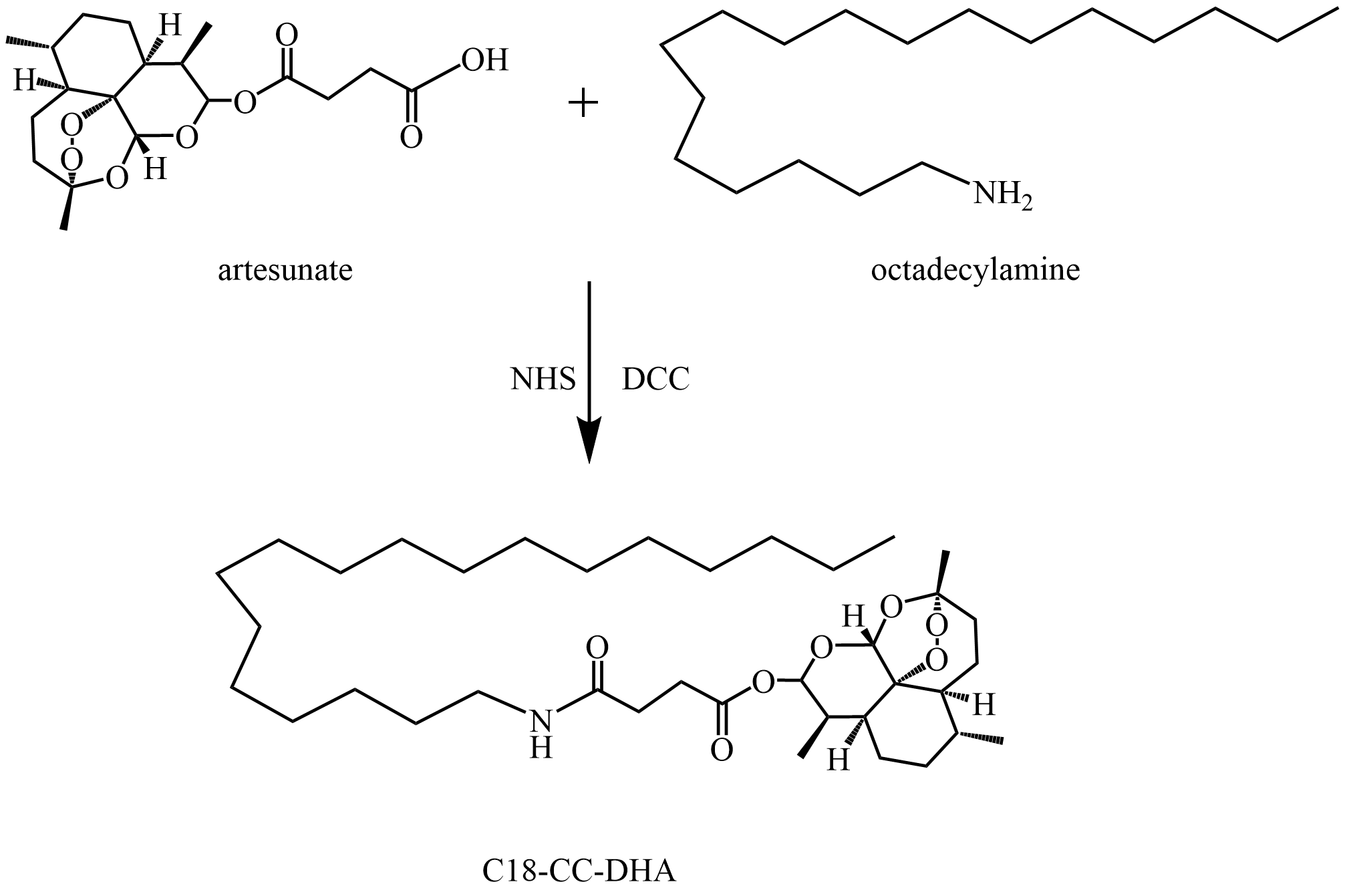


Fig. S6. Synthetic scheme of C18-CC-DHA.

Fig.S7. FTIR of C18-CC-DHA.


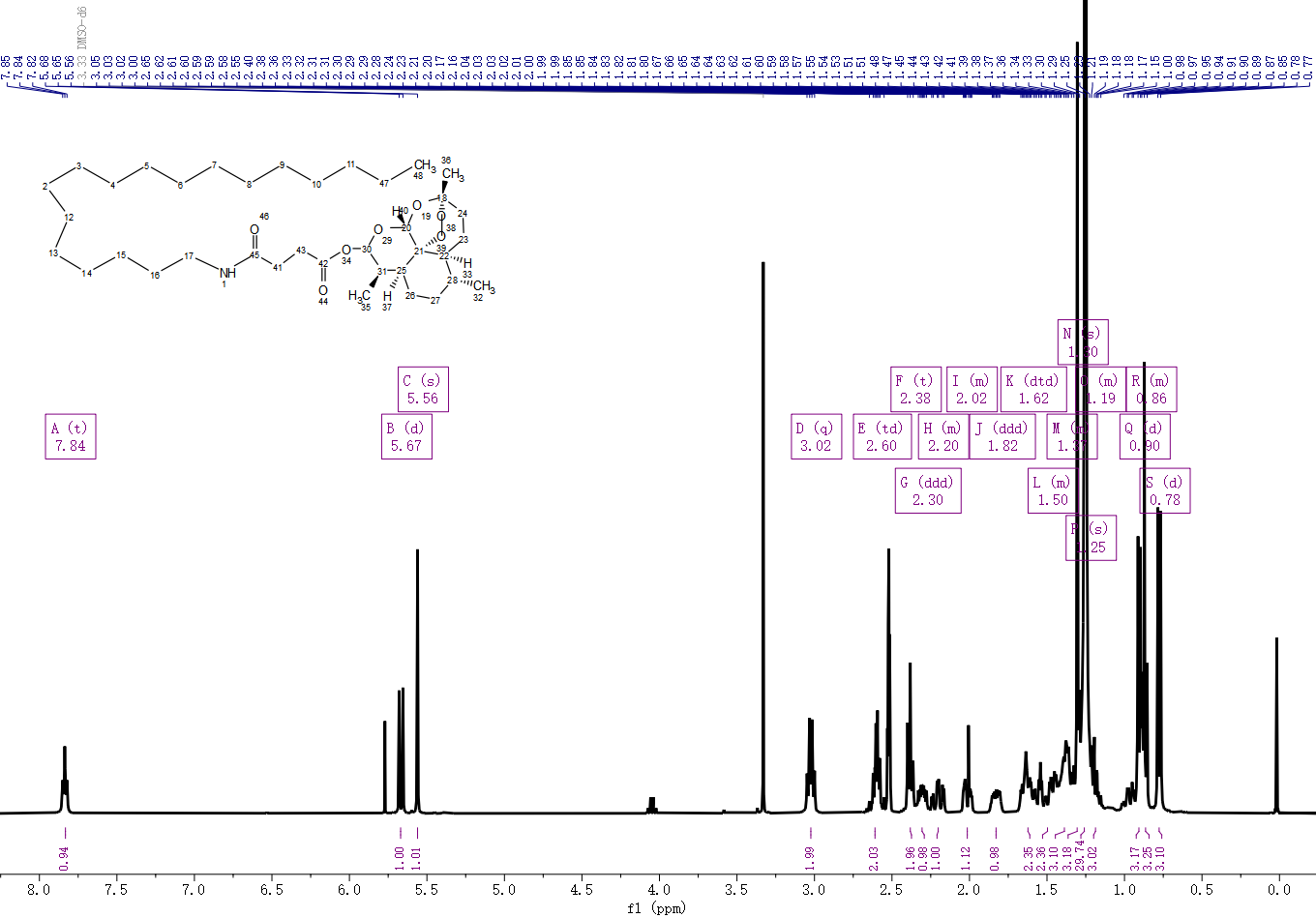


Fig. S8. ^1^H NMR of C18-CC-DHA.

Fig. S9. ^13^C NMR of C18-CC-DHA.

Fig. S10. HR-MS of C18-CC-DHA.

Fig. S11. Synthetic scheme of TPGS-GLU.

Fig. S12. ^1^H NMR of TPGS-COOH.

Fig. S13. FTIR of TPGS-GLU.

Fig. S14. ^1^H NMR of TPGS-GLU.

Fig. S15. ^13^C NMR of TPGS-GLU.


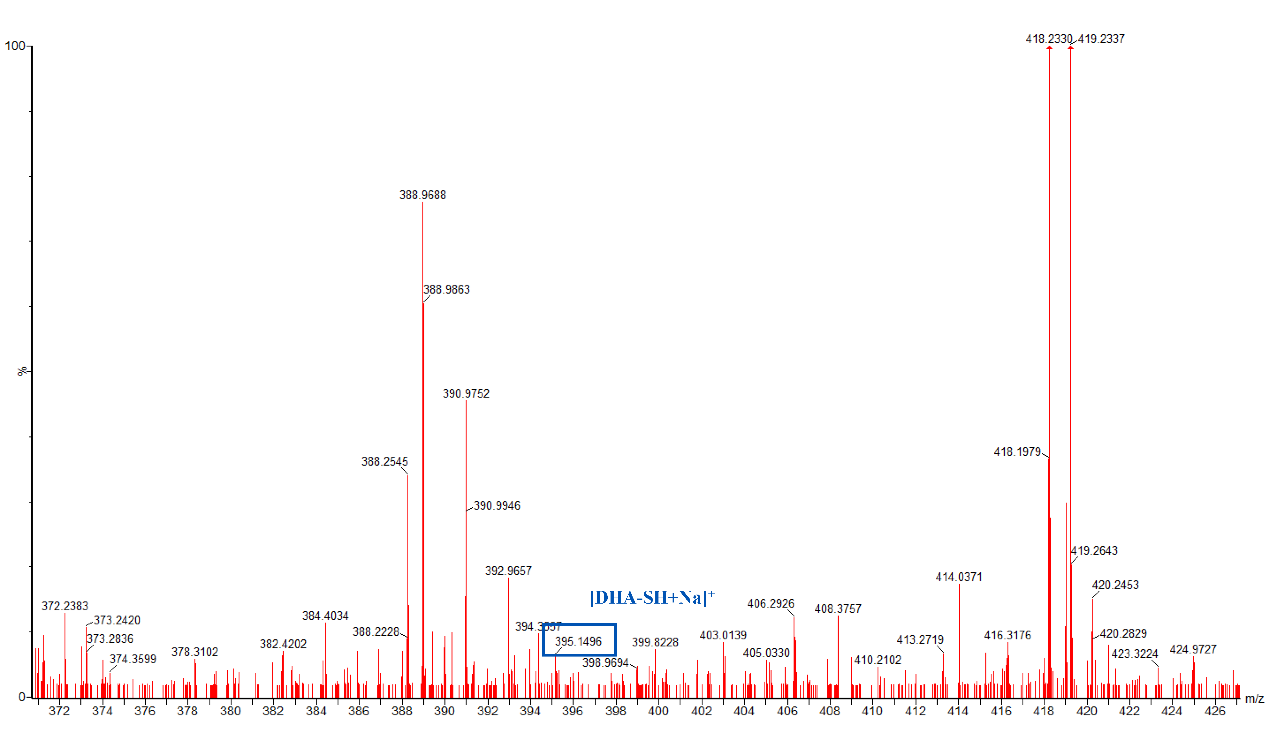


Fig. S16. Mass spectra of D@GLU-PMs-SS after incubated with 5 mM GSH-containing release medium.


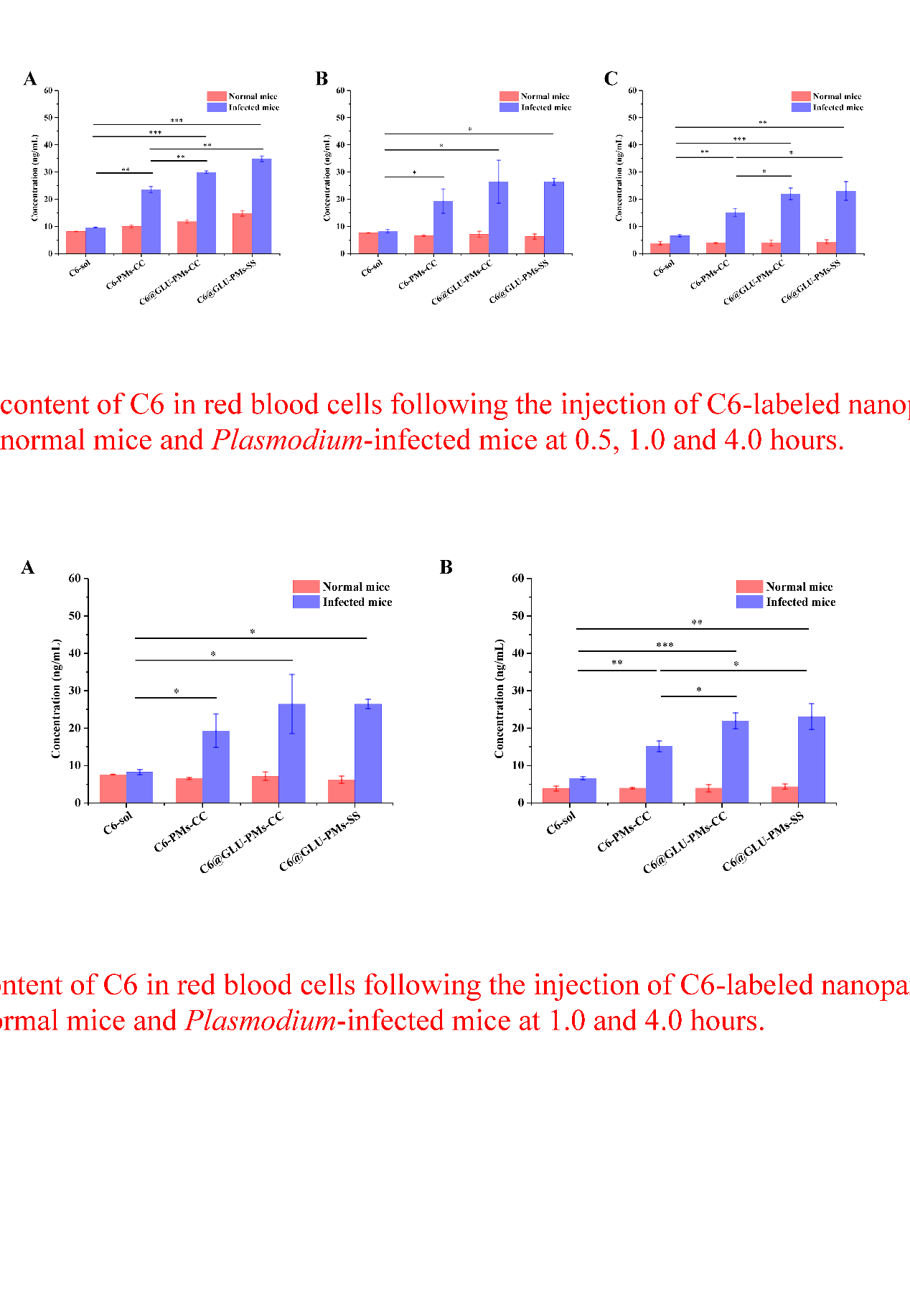


Fig. S17. The fluorescence intensity in red blood cells after injecting C6-PMs-CC, C6@GLU-PMs-CC and C6@GLU-PMs-SS into normal mice and Plasmodium-infected mice. A: 1.0 hours after injection, B: 4.0 hours after injection. The data are presented as means ± SD and were analyzed by independent samples t -test (^*^*P* < 0.05, ^**^*P* < 0.01, ^***^*P* < 0.001, n = 3).

Table S1. Size, PDI, and Zeta Potential of D-PMs-CC, D@GLU-PMs-CC and D@GLU-PMs-SS (*n* = 3).

| Sample | Size (nm) | PDI | Zeta potential (mV) |
| --- | --- | --- | --- |
| D-PMs-CC | 157.50 ± 5.74 | 0.13 ± 0.05 | -18.93 ± 1.40 |
| D@GLU-PMs-CC | 165.40 ± 8.20 | 0.14 ± 0.02 | -20.97 ± 1.22 |
| D@GLU-PMs-SS | 161.70 ± 3.61 | 0.11 ± 0.03 | -10.09 ± 0.81 |

Table S2. Encapsulation efffciency and Drug-Loading efffciency of D-PMs-CC, D@GLU-PMs-CC and D@GLU-PMs-SS (*n* = 3).

| Sample | EE (%) | DL (%) |
| --- | --- | --- |
| D-PMs-CC | 93.92 ± 0.49 | 85.38 ± 0.44 |
| D@GLU-PMs-CC | 94.81 ± 1.18 | 86.19 ± 1.07 |
| D@GLU-PMs-SS | 97.86 ± 0.47 | 88.96 ± 0.43 |

Reference

Wang, R., Ren, G., Wang, R., Zhang, L., Zhang, S., 2019. Preparation and antimalarial activity of octadecylamine-modified dihydroartemisinin prodrug self-assembled nanoparticles. Chinese Journal of Pharmaceuticals 50, 1201-1207.<https://doi.org/10.16522/j.cnki.cjph.2019.10.013>.
